# Listeria Monocytogenes – characterization of strains isolated from clinical severe cases

**Published:** 2014

**Authors:** AM Borcan, S Huhulescu, A Munteanu, A Rafila

**Affiliations:** * ”Matei Bals” National Institute for Infectious Diseases, Bucharest, Romania; ** Austrian Agency for Health and Food Safety (AGES), Vienna, Austria; *** National Institute for Public Health, Bucharest, Romania

**Keywords:** Listeria monocytogenes, strains, subtyping, serotyping, severe clinical infection

## Abstract

*Listeria monocytogenes* became an increasing pathogen involved more frequently in sporadic severe illnesses and outbreaks of foodborne infections.

This study investigates in vitro susceptibility of 26 strains of *Listeria monocytogenes* isolated from the clinical specimens collected between March 2009 and November 2013, from 24 patients hospitalized in three medical institutions in Bucharest.

All isolates were tested by disk diffusion method to 15 antimicrobial agents, by using disk diffusion tests. Among the 26 clinical *L. monocytogenes* isolates tested, no multidrug resistant strains were detected, but 18 (72%) were found to be resistant to at least one clinically relevant antibiotic. Among them, 18 clinical isolates were resistant against ciprofloxacin also. Resistance to Ciprofloxacin was particularly noticed to the strains in Romania.

Serological and molecular typing by Multiplex PCR method detected two molecular types 1/2 a, 3a and 1/2 b, 3b, as to the more frequent isolated among studied cases. These types of *L. monocytogenes* could be associated to the higher pathogenic activity of immunodeficient patients.

## Introduction

Of the ten currently known Listeria (L.) species (*L. monocytogenes, L. ivanovii, L. Seeliger, L. Innocua, L. welshimeri, L. gray* (McLauchlin, 2005), *L. rocourtiae* (Leclerq et al., 2010), *L. fleischmanii* (Bertsch et al., 2013) and *L. weihenstephanensis, L. monocytogenes* is almost exclusively responsible for human diseases [**1**].

Listeriosis, a common foodborne disease can become a severe illness in high risk populations, such as pregnant women or newborns, and patients with underlying immunodeficient diseases. Listeriosis can develop an invasive nature and can lead to high lethality (20-30%) [**2**] in cases of immunodeficient patients. Due to the ubiquitous presence of *L. monocytogenes*, the risk of infection can be represented by a large variety of animal and vegetable food products [**3**].

*L. monocytogenes* is considered a hazardous agent in the food industry, due to its ability to grow and multiply at low temperature or gas or in products stored and kept refrigerated, being able to multiply at refrigeration temperatures and to form biofilms [**3**,**4**].

*L. monocytogenes* also survive in different combinations of pH and salt concentrations and develop resistance to heavy metals or sanitizers [**5**] used in the food industry. In contrast to other bacteria, *L. monocytogenes* has not been developed as a high resistant organism to antibiotics commonly used in the therapy of infection diseases [**6**]. However, the first strains of *L. monocytogenes* showing a multidrug-resistant [**7**-**10**] characteristic have been described in the years 1988-1995. Morvan et al. [**11**] analyzed the susceptibility to 27 antibiotics of 4,816 clinical *L. monocytogenes* strains, isolated between 1926 and 2007, and found that resistance against tetracyclines and fluoroquinolones were more common and has recently emerged. In our work, we investigated the serologic and molecular types as well as the in vitro resistance profile of 26 *L. monocytogenes* strains isolated from the clinical specimens collected from severe cases hospitalized between March 2009 - November 2013. 

## Materials and methods

**Cases**

A total of 26 strains of L. monocytogenes from 20 patients hospitalized between March 2009 and November 2013, in three medical institutions in Bucharest: Babes Hospital, Elias Hospital, “Matei Bals” National Institute of Infectious Diseases was available for study. Five patients showed meningoencephalitis, three septicaemia (one of them a new born), two meningoencephalitis plus septicaemia, three meningitis and septicaemia. Three cases were pregnancy associated; one isolated originating from the blood of a new-born (**Table 1**).

24 confirmed cases, predominantly urban residence (6:1), female sex (2:1), age group "15-64" (64%).

The cases were classified in risk groups such as invasive disease: newborn - 4%; Pregnant - 8% and comorbidities were accompanied by a degree of immunodeficiency - 62%, 92% of the transmission were probably the food, the vehicle detected in any case; 4% vertical transmission and possible food 4% transmission associated with professional contact with birds.

The origin of the 26 isolated cases of *L. monocytogenes* was the following: CSF-16; blood-culture – 9; placenta swab – 1.

**Table 1:** Origin of the 26 clinical isolates of tested *L. monocytogenes*

Origin of isolated strains	No. of strains
CSF	16
Blood-culture	9
Placenta swab	1
Total	26

**Studied L. monocytogenes isolates**

**Laboratory isolation.** The specimens were routinely investigated in “Matei Bals” National Institute of Infectious Diseases laboratory according to recommendations of Alerberger [**7**] and by using ready for use media and reagents delivered by Sanimed International Impex Srl.

**Phenotyping investigation.** Enzymatic characterization and serological typing of all isolated strains was performed in National Reference Center for Zoonotic Infections in “Cantacuzino” National Institute of Research-Development for Microbiology and Immunology, according to Caplan’s recommendations [**31**].

**Serotyping.** The strains were serotyped in the same National Centre by slide agglutination test by using in house prepared rabbit sera.

**Detection of amylase activity** was performed by spotting isolates onto agar with 1% starch as substrate (medium prepared in house). After 72h incubation period at 37°C, the starch hydrolysis appeared as a precipitation zone surrounding the culture spot.

**Detection of DNAse** production was performed by spotting isolates onto DNA agar medium (medium prepared in house). After incubation for 24h period at 37°C, a drop of HCl 1N solution was added to each spotted culture and the reactions were examined. A clearing area surrounding the culture spot area is considered a positive reaction.

**Detection of esculinase activity** was performed by spotting isolates onto agar plates with 1% esculin (medium prepared in house), followed by incubation for 18 h at 36±2°C in an ambient atmosphere. Esculin hydrolysis results in black coloration of the medium.

**Molecular investigations**

Were performed at the Austrian Reference Center for Listeria in Vienna, Austria. Species diagnosis was confirmed by using API-Liseria (bioMérieux, Marcy l’Etoile, France) and mass spectrometry (MALDI-TOF Biotyper; Bruker Daltonics, Bremen, Germany).

All isolates were serotyped by multiplex PCR as described by Doumith et al [**12**] and subtyped by HRM-PCR-Analysis [**13**] and by pulsed-field gel electrophoresis [**14**]. High resolution melting curve-PCR (HRM-PCR) analysis was performed as described by Pietzka et al. [**13**]. Briefly, genomic bacterial DNA (gDNA) is extracted from bacterial cells grown overnight at 37°C on blood agar (bioMérieux, Marcy l’Etoile, France) by using the QuickExtractTM DNA extraction solution (Epicentre Biotechnologies, Madison, USA) according to the manufacturer’s instructions. Purified gDNA is quantified spectrophotometrically at 260 nm and the gDNA quality assessed by the 260/280 ratio method [**15**]. A 500-bp fragment located in the virulence gene internalin B (*inlB*) is amplified for subsequent HRM analysis by using the forward primer *inlB* -forward(5’-CATGGGAGAGTAACCCAACC-3’) and the reverse primer *inlB*-reverse (5’-GCGGTAACCCCTTTGTCATA-3’) [**16**].

PFGE was performed according to the standardized PulseNet protocol for molecular subtyping of *L. monocytogenes* by pulsed-field gel electrophoresis (PFGE) [**14**]. Clusters were defined as two or more isolates which exhibit an indistinguishable PFGE patter in both enzymes. Gels were photographed under UV-transillumination by using a GelDoc2000 documentation system (Bio-Rad, Vienna, Austria) and patterns were analyzed by using bionumerics software 6.6 (Applied Maths, Saint_Martems-Latem, Belgium) and the following settings: 0,5% optimization, 0.8 position tolerance, dice similarity coefficient.

**Pathogenic investigations.**

**Bacterial adherence to HEp-2 cells**

Adherence tests were performed at the Austrian Reference Centre for Listeria in Vienna, also by using HEp-2 cells ATCC CCL-23 (ATCC, Manassas, USA) as described by Cravioto et al. [**17**]. Briefly, cells were cultured in Eagle minimum essential medium (EMEM) (Irvine Scientific, Santa Anna, CA).

**Detection of virulence factors**

-*Detection of enhancement of hemolysis* was performed by Christie, Atkins, Munch and Petersen (CAMP) test as recommended by ISO and the association of official analytical chemists (AOAC) protocols.

-*Detection of lecithinase and lipase* activity was performed by spotting isolates onto 2.5% yolk agar and onto 1% Tween-80 agar (both media prepared in house). A clearing zone surrounding the growth area after 72h incubation at 37°C was considered a positive reaction for lecithinase activity, and an opaque (precipitation) area a positive reaction for lipase activity.

-*Detection of caseinase and gelatinase* activity was performed by spotting isolates onto agar plates with 15% soluble casein (medium prepared in house), or gelatine plates at 1% final concentration (medium prepared in house). After incubation at 37°C for 72h, proteolytic activity is reflected by an area of clarification around the spot culture.

**Antimicrobial sensitivity testing (AST)**

AST by standard disk diffusion method was performed according to the Clinical and Laboratory Standard Institute (CLSI) – standard reference procedures 2012 [**18**] by using antibiotic impregnated standard disks Oxoid (Oxoid Basingstoke, UK) and Mueller Hinton agar (Merck Dornstad Germany). *L. monocytogenes* ATCC 1911 and *Staphylococcus aureus*, ATCC 25923 strains were used as reference strains. The plates were incubated aerobically for 24 hrs at 35°C. The inhibition area was interpreted by CLSI guide for non fastidious microorganisms, but “intermediate susceptible” strains were coated as “resistant” in our study.

## Results

All 26 isolates demonstrated similar phenotypic behavior concerning their cultivability and exoenzymatic activity being identified as typical Listeria monocytogenes species.

Molecular and antigenic characterization of 26 strains is presented in **Table 2**. Serotyping by in house agglutinating sera has shown the existence of two serotypes only: 1a (dominant – 22 strains) and 4b encountered in four strains (2 from CSF and 2 from blood).

Serotyping by Multiplex PCR classified the 26 isolates in 3 serovars: 1/2a, 3a (15 strains), 4b, 4d, 4e (10 strains) and 1/2c, 3c (1 strain) (**Table 2**).

**Table 2:** Molecular and antigenic characterization of *L. monocytogenes* strains

								ID-No.							
		C1	C2	C3	C4	C5	C6	C7	C8	C9	C10	C11	C12a	C12b	C13
Molecular antigens	HRM-PCR(InIB ST)	ST-2	ST-16	ST-2	ST-15	ST-2	ST-30	ST-30	ST-9	ST-9	ST-2	ST-1	ST-15	ST-15	ST-15
	Typing by Multiplex PCR	4b,4d,4e	1/2c,3c	4b,4d,4e	1/2a,3a	1/2a,3a	1/2a,3a	1/2a,3a	1/2a,3a	1/2a,3a	4b,4d,4e	4b,4d,4e	1/2a,3a	1/2a,3a	1/2a,3a
	Serotyping by in house agglutination	4b	1a	1a	1a	4b	1a	1a	1a	1a	4b	1a	1a	1a	1a
								ID-No.							
		C14	C15	C16	C17	C18	C19	C20	C21	C22	C23	C24	C25	C26
Molecular antigens	HRM-PCR(InIB ST)	ST-15	ST-1	ST-8	ST-2	ST-1	ST-1	ST-8	ST-2	ST-15	ST-30	ST-9	ST-8	ST-16
	Typing by Multiplex PCR	1/2a,3a	4b,4d,4e	1/2a,3a	4b,4d,4e	4b,4d,4e	4b,4d,4e	1/2a,3a	4b,4d,4e	1/2a,3a	1/2a,3a	1/2a,3a	1/2a,3a	1/2a,3a
	Serotyping by in house agglutination	1a	4b	1a	1a	1a	1a	1a	1a	1a	1a	1a	1a	1a
Summarized results of 26 human isolates (including two isolate-pairs from two patients and one mother/child pair). C: human isolates; C12a, C12b: mother/child pair; C6/C7: CSF/blood isolate pair; C8/ C9: CSF/blood isolate pair; InlB ST: Internalin B sequence type.

Subtyping by HRM-PCR Analysis showed 7 distinct internalin B (Int B) sequence types (ST3).

Five strains were wrongly included in serotype 1a by in house sera agglutination. By multiplex PCR method it was proved to be 4b, 4d, 4e type.

While in serovar 4b, 4d, 4e, two subtypes ST1 and ST2 were identified, in serovar 1/2a, 3a, 4 sequence types ST8, ST9, ST15 and ST30 were differentiated. In subtype ST15 6 strains were included; four isolated from CFS, 1 from blood and 1 from placenta (the last two from the same patient).

The characterization of bacterial adherence to HEp2 cells and the presence of virulence factors of 15 Listeria strains is listed in **Table 3** (7 strains not tested).

**Table 3:** Bacterial adherence to Hep-2 cells and virulence factors

							ID-No.							
	C1	C2	C3	C4	C5	C6	C7	C8	C9	C10	C11	C12a	C12b	C13
Specimen	CSF	CSF	CSF	CSF	Blood	CSF	Blood	CSF	Blood	CSF	CSF	Blood	Placental swab	CSF
**Adherence factor **(% of bacterial cells adhering)	nd	nd	nd	nd	Localized aggregative 20%	Diffuse 83%	No adherence	Diffuse 95%	Localized aggregative 20%	nd	Localized aggregative 8%	nd	nd	Diffuse 22%
Virulence factors	nd	nd	nd	nd	h,e,c	h,e,c	h,e,c	h,e,c	h,e,c	nd	h,e,c	nd	nd	h,e,c
							ID-No.							
	C14	C15	C16	C17	C18	C19	C20	C21	C22	C23	C24	C25	C26
Specimen	CSF	Blood	CSF	CSF	CSF	CSF	CSF	CSF	CSF	CSF	Blood	CSF	CSF
**Adherence factor **(% of bacterial cells adhering)	No adherence	Localized aggregative 5%	Localized aggregative 10%	No adherence	Diffuse 20%	Diffuse 5%	Localized aggregative 3%	Diffuse 20%	Localized aggregative 10%	No adherence	Diffuse 22%	No adherence	Localized aggregative 5%
Virulence factors	h,e,c	h,e,c	h,e,c	h,e,c	h,e,c	h,e,c	h,e,c	h,e,c	h,e,c	h,e,c	h,e,c	h,e,c	h,e,c
h: hemolysin; e: esculinase; c: caseinase; li: lipase; nd: not done; CSF: cerebrospinal fluid; **: pregnancy associated; +/-: weak activity

Four human isolates showed lack of adherence. The other strains exhibited two adherence patterns at various levels: localized aggregative (graded from 3% to 20%) - 6 strains and diffuse (graded as 5% to 95%) – 7 strains. All isolates which showed diffuse adherence originating from CFS: 13 belonged to serovar 1/2a, 3a and 3 to 4b, 4d, 4e.

Three CSF isolates (both serovar 1/2a, 3a) demonstrated no adherence in vitro.

All 15 isolates tested for the presence of virulence factors, demonstrated production of hemolysin, esculinase and caseinase.

The resistance to antibiotics of 26 isolates is summarized in **Table 4**. All strains were resistant to cefuroxime and nalidixic acid.

Eighteen clinical isolates were resistant in addition to ciprofloxacin and two, to ampicillin and amoxicillin clavulanate. Both strains belonged to the 1/2a, 3a serotype. Only one strain was resistant to erythromycin. No resistance strain was encountered to the other 7 current used antibiotics. There was no relationship in between antibiotic resistance pattern and the serovar of the strains.

**Table 4:** Antibiotic resistance of 26 *L. monocytogenes* clinical isolates

			Antibiotic resistance					
	*Cefuroxime*	*Nalidixic acid*	*Ciprofloxacin*	*Ampicilin*	*Amoxicillin-clavulanate*	*Imipenem*	*Penicilin*	*Erythromycin*
Number of strains	26	26	18	2	2	2	2	1
Serotype	1/2a, 3a 4b,4d,4e	1/2a, 3a 4b,4d,4e	4b,4d,4e 1/2a, 3a	1/2a, 3a	1/2a, 3a	1/2a, 3a	1/2a, 3a	4b,4d,4e

**Comments**

*L. monocytogenes* can cause listeriosis, a rare but serious infection, which shows a high case-fatality ratio [**1**-**4**]. A lethality rate of 35% observed for our Romanian patients, underlines the public health significance of this foodborne illness.

Serotyping was the first subtyping method developed for *L. monocytogenes* [**19**]. In epidemiological investigations, bacterial serotyping is usually unable to estimate the relatedness of different isolates, as invasive listeriosis is caused mostly by serotypes 1/2 a, 1/2b and 4b strains. Therefore, serotyping of* L. monocytogenes* has only limited practical value for the investigation of the chains of transmission.

Furthermore, there are no standardized protocols for the preparation of rabbit sera. Most reference laboratories today rely on a PCR based serotyping method, developed by Doumith et al. [**12**]. Their multiplex PCR divides *L. monocytogenes* isolates into four groups employing primers annealing to Listeria genus specific prs and genes specific to serotype-associated phylogenetic lineages of *L. monocytogenes*. This method can differentiate between strains of serotypes “1/2a, 3a”, “1/2c, 3c”, “1/2b, 3b” and the serotypes “4b, 4d, 4e” [**12**]. Our study revealed a high rate of discordant results between data obtained by classical serotyping and multiplex PCR. Testing of bacterial adhesion to HEp-2 cells is used as a parameter to assess virulence of *L. monocytogenes* isolates [**20**]. In our study, we were unable to correlate adhesion patterns to virulence: neither fatal outcome nor source of isolates correlated with the adherence patterns. There was even no concordance of adherence patterns between the epidemiologically related specimen strains C6/C7 and C8/C9.

Recently, Moroni et al. postulated the absence of correlation between the adhesion and invasion level [**21**].

Hemolysin, lipase and lecithinase are important virulence factors in listeriosis [**22**-**24**]. The loss of hemolysin production in mutant strains results in avirulence [**25**]. Lipase and lecithinase are implicated in pore forming and bacterial invasion [**22**]. Caseinase and gelatinase foster tissue damage and rapid bacterial multiplication [**26**]. Amylase is implicated in polysaccharide hydrolysis, offering a competitive nutritional advantage to bacterial strains producing this enzyme [**27**,**28**].

In our study, all but one isolates of *L. monocytogenes* were sensitive to beta-lactam antibiotics (ampicillin, amoxicillin, imipenem) and gentamicin associations of these two groups of antibiotics were currently used as therapeutic choice in *L. monocytogenes* infections. But one strain isolated from the same patients (from hemoculture and CSF) demonstrated in vitro resistance to the above mentioned beta lactams suggesting a reconsideration of therapy after antibiotic testing.

Taking into account that *L. monocytogenes* is intrinsically resistant against cefuroxime and nalidixic acid, we noticed a developing resistance to ciprofloxacines, frequently among our studied strains (half of all the strains) particularly.

In 2008, Morobe et al. [**29**] found that the most common resistance among food isolates was against penicillin (45%), followed by tetracycline (32,2%), while Wong et al. [**30**] showed that resistance to tetracycline was the most common - 46,3% - followed by erythromycin 36,6%, amikacin 31,7% and sulfamethoxazole – trimethoprim 17,1%.

Morovan et al. [**11**] also pointed in an extended study that resistance against fluoroquinolones was more common and has become an alarming phenomenon.

## Conclusions

Our study performed on 26 *L. monocytogenes* strains isolated from specimens of patients with severe clinical infections (20 CSF, 5 hemocultures and septic abortion) showed that:

1. Two serotypes types “1a”-dominant (18 strains) and “4b” (4 strains) suggesting these types to be more often encountered in severe clinical infection determined by *L. monocytogenes*.

2. Serotyping by Multiplex PCR system confirmed the 1a belonging type, except for 4 strains which were identified as “b” type (4b, 4d, 4e). HRM-PCR Analysis could be admitted as a supplementary system useful in the epidemiological investigation (no clinical significance).

3. Hemolysin, lipase and lecithinase are important virulence factors easy to perform in listeriosis. Bacterial adhesion to HEp-2 cells may be adopted as a parameter to assess virulence of *L. monocytogenes* strains.

4. Antibiotic testing by disk diffusion method demonstrated that:

- Multidrug resistance was not detected among our strains

- Resistance to cefuroxime and nalidixic acid was intrinsic species property as the other authors pointed. Unexpectedly, more than half of the isolates (18/26) were resistant to ciprofloxacin. This resistance was particularly noticed to our strains.

- Two strains (from the same patient) were resistant to ampicillin and amoxicillin-clavulanate, so we suggest a reconsideration of the policy of primary antibiotherapy in listeriosis. AST is recommended in listeriosis, too.
